# Construction of an Immobilized Thermophilic Esterase on Epoxy Support for Poly(ε-caprolactone) Synthesis

**DOI:** 10.3390/molecules21060796

**Published:** 2016-06-18

**Authors:** Hui Ren, Zhen Xing, Jiebing Yang, Wei Jiang, Gang Zhang, Jun Tang, Quanshun Li

**Affiliations:** 1 Department of Colorectal Surgery, the Second Hospital of Jilin University, Changchun 130041, China; hren@jlu.edu.cn; 2 Key Laboratory for Molecular Enzymology and Engineering of Ministry of Education, School of Life Sciences, Jilin University, Changchun 130012, China; xingzhen14@mails.jlu.edu.cn (Z.X.); yjb13804460320@sina.com (J.Y.); 3 Department of Polymer Science, College of Chemistry, Jilin University, Changchun 130012, China; jiangw0909215@163.com

**Keywords:** thermophilic esterase, immobilization, epoxy support, ring-opening polymerization, poly(ε-caprolactone)

## Abstract

Developing an efficient immobilized enzyme is of great significance for improving the operational stability of enzymes in poly(ε-caprolactone) synthesis. In this paper, a thermophilic esterase AFEST from the archaeon *Archaeoglobus fulgidus* was successfully immobilized on the epoxy support Sepabeads EC-EP *via* covalent attachment, and the immobilized enzyme was then employed as a biocatalyst for poly(ε-caprolactone) synthesis. The enzyme loading and recovered activity of immobilized enzyme was measured to be 72 mg/g and 10.4 U/mg using *p*-nitrophenyl caprylate as the substrate at 80 °C, respectively. Through the optimization of reaction conditions (enzyme concentration, temperature, reaction time and medium), poly(ε-caprolactone) was obtained with 100% monomer conversion and low number-average molecular weight (*M*_n_ < 1300 g/mol). Further, the immobilized enzyme exhibited excellent reusability, with monomer conversion values exceeding 75% during 15 batch reactions. Finally, poly(ε-caprolactone) was enzymatically synthesized with an isolated yield of 75% and *M*_n_ value of 3005 g/mol in a gram-scale reaction.

## 1. Introduction

Poly(ε-caprolactone) (PCL) is an important type of biomaterial with excellent characteristics, such as good degradability, biocompatibility and histocompatibility. It has been widely used in several fields of biomedical engineering including long-term drug delivery, tissue engineering, microelectronics and surgical suturing [1]. In the past two decades, PCL synthesis using lipases or esterases as catalysts have been extensively studied [2,3,4,5,6] as a potential alternative to chemical routes. Compared to chemical strategies, enzymatic PCL synthesis possessed many advantages [7]: (1) mild reaction conditions; (2) fewer by-products and (3) absence of trace metallic catalyst residues, especially for biomedical applications. In addition, construction of polymers with unique structures or properties based on PCL segments could be achieved by the combination of enzymatic polymerization and chemical routes [8], e.g., block copolymers synthesized by combining ring-opening metathesis polymerization and enzymatic ring-opening polymerization [9,10]. 

Many lipases or esterases have been successfully used as catalysts for PCL synthesis, such as porcine pancreatic lipase, *Pseudomonas cepacia* lipase, *Candida antarctica* lipase A or B and *Humicola insolens* cutinase [11,12,13,14,15]. However, due to their low stability under harsh reaction conditions (high temperature and exposure to organic solvents), these enzymes are not well suited for the enzymatic synthesis of PCL. To solve the obstacle, it is of great significance to explore enzymes with high stability under extreme conditions [16]. Compared to mesophilic enzymes, enzymes from thermophiles have been considered as potential catalysts in industrial biocatalysis and biotransformation due to their excellent stability against high temperature, organic solvents or detergents. In addition, thermophilic enzymes could perform faster reactions and be easily separated from other heat-labile proteins during purification steps [17,18]. In our previous research, a thermophilic esterase AFEST from *Archaeoglobus fulgidus* has been demonstrated to possess promising PCL synthesis activity, with *ca.* 100% monomer conversion and number-average molecular weight (*M*_n_) of 1400 g/mol [19]. 

Compared with free enzymes, enzyme immobilization is an efficient route for improving the biocatalytic process economics by enzyme reuse and the enhancement of enzyme stability, thereby permitting catalysis under harsh reaction conditions on an industrial scale [20,21,22,23]. Meanwhile, enzyme immobilization has also been successfully used to optimize the performance of a series of thermophilic enzymes, including lipases and esterases [24,25]. Among the carriers, Sepabeads, developed by Resindion, have been widely used in enzyme immobilization, either through physical adsorption or covalent attachment [26,27]. In our previous research, hydrophobic Octadecyl-Sepabeads EC-OD macroporous resin was employed as a carrier for constructing immobilized enzymes through physical adsorption to realize the repeated use of the biocatalysts and reduce the cost in PCL synthesis [28]. However, after reusing the immobilized enzyme for five cycles, only 36% of its original activity was retained, probably due to the loss of adsorbed enzyme from the carrier during consecutive reactions [29]. Thus, it is urgent to develop a more efficient immobilized enzyme for improving the reusability in PCL synthesis, e.g., through covalent attachment [30,31]. As epoxy supports could react with different nucleophilic groups on the protein surface (e.g., amino, hydroxy, or thiol moieties) to form extremely strong linkages with minimal chemical modification of the protein, epoxy-activated supports could be ideal systems for constructing immobilized enzymes [32,33,34,35,36,37]. In addition, epoxy groups are very stable at neutral pH values and the supports could be stored for a long period [33,34]. 

In the present research, the covalent attachment of thermophilic esterase AFEST on an epoxy support and its application in polyester synthesis were systematically investigated, using the ring-opening polymerization of ε-caprolactone as a model. Effects of reaction conditions on monomer conversion and product molecular weight were evaluated to get deeper insight into the differences between free enzyme and immobilized enzyme. Furthermore, an operational stability assay was employed to assess the recyclability of the immobilized enzyme. 

## 2. Results and Discussion

### 2.1. Immobilization of Thermophilic Esterase on Epoxy-Sepabeads EC-EP

**Figure 1:**
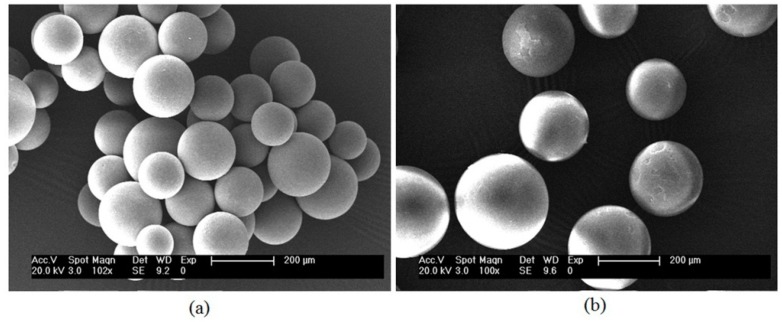
SEM analysis of surface morphologies of Sepabeads EC-EP support (**a**) and immobilized enzyme EC-EP-AFEST (**b**).

According to our previous report [19], thermophilic esterase AFEST was purified with an active protein content of 92% (*w*/*w*) of solid powder, with a specific activity of *ca.* 168 U/mg determined by using *p*-nitrophenyl caprylate as the substrate at 80 °C. Then the immobilization of thermophilic esterase AFEST (1 mg/mL) was performed on Epoxy-Sepabeads EC-EP. Generally, enzyme immobilization on epoxy supports consists of two stages [38]: rapid physical adsorption and subsequent covalent attachment between the epoxy groups of the support and amine groups of the enzyme. The covalent attachment between the soluble enzyme and epoxy supports is usually very slow, and alkaline conditions are favorable for the reaction progress [38,39,40,41]. Thus, in the present research 50 mM phosphate buffer (pH 8.0) was selected to facilitate the covalent attachment. After enzyme immobilization, enzyme loading and recovered activity were calculated to be 72 mg AFEST/g support and 10.4 U/mg immobilized enzyme by monitoring the enzymatic activity, respectively. The surface morphologies of carriers and immobilized enzymes were studied using SEM analysis, as shown in Figure 1. In contrast to the smooth surface of the carrier, the immobilized enzymes possessed a rough layer and exhibited a greater particle size after immobilization, indicating the successful immobilization of enzyme on the Sepabeads EC-EP epoxy supports. 

### 2.2. Enzymatic Synthesis of PCL Using Immobilized Enzyme EC-EP-AFEST

Through ^1^H-NMR characterization, we confirmed the structure of PCL synthesized by EC-EP-AFEST, which was consistent with our previous report [19]. In the enzymatic polymerization, the reaction conditions, including enzyme concentration, temperature, reaction time and medium, play an important role in the monomer conversion and product molecular weight. Thus, similar to the free enzyme, we investigated the effects of reaction conditions to improve the monomer conversion and product molecular weight, while discriminating the differences between free enzyme and its immobilized form. 

**Figure 2:**
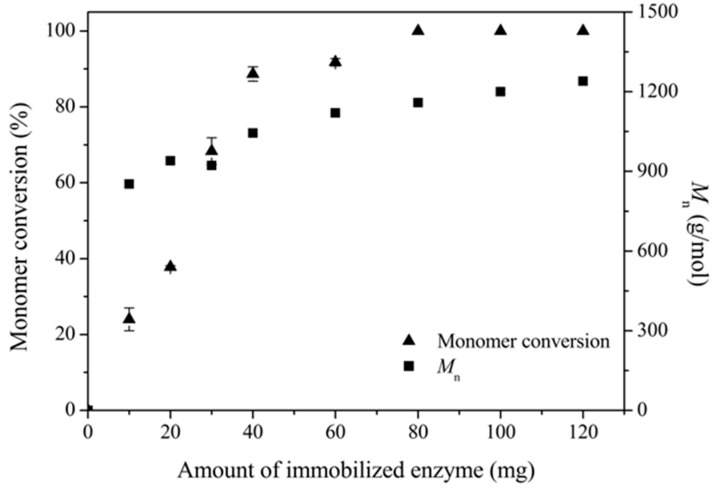
Monomer conversion (▲) and *M*_n_ (■) as a function of the amount of immobilized enzyme EC-EP-AFEST in the ring-opening polymerization of ε-caprolactone. The reactions were carried out using 200 μL ε-caprolactone and 600 μL toluene at 80 °C for 72 h.

The effect of enzyme concentration was investigated first using different amounts of immobilized enzyme, 200 μL ε-caprolactone and 600 μL toluene at 80 °C for 72 h. It was obvious that both the monomer conversion and *M*_n_ values were improved with an increase in enzyme concentration (Figure 2). 

When the amount of enzyme in the reaction system exceeded 80 mg, the monomers were completely converted into polymeric chains. Nevertheless, *M*_n_ values remained in the 850–1240 g/mol range. Notably, the products showed narrow molecular weight distributions with polydispersity indexes (PDI) lower than 1.30. Thus, PCL obtained in the present research was of low molecular weight (much lower than 44,800 g/mol produced by immobilized *C. antarctica* lipase B [11]), and could potentially be used as a soft segment in polyurethane or a localized drug delivery system. The immobilized enzymes displayed the same catalytic behavior as free enzyme, and the immobilization did not affect the nature or characteristics of the enzyme. From the viewpoint of monomer conversion and *M*_n_, 80 mg EC-EP-AFEST was employed in the subsequent research.

**Figure 3:**
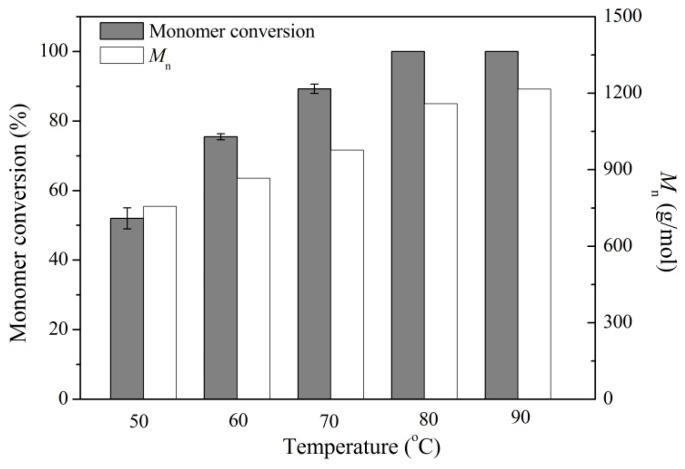
Effect of temperature on monomer conversion and product molecular weight *M*_n_. The reactions were carried out using 200 μL ε-caprolactone and 600 μL toluene at different temperatures for 72 h.

**Figure 4:**
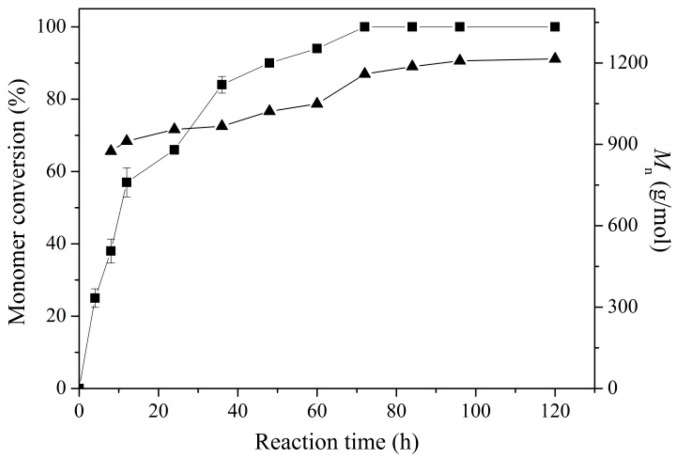
Monomer conversion (▲) and *M*_n_ (■) as a function of reaction time. The reactions were carried out using 80 mg EC-EP-AFEST, 200 μL ε-caprolactone and 600 μL toluene at 80 °C for different reaction times.

Next we assessed the effect of reaction temperature on monomer conversion and product molecular weight. As shown in Figure 3, high temperature was favorable for the progress of the polymerization reactions due to the enzyme’s high catalytic activity at high temperature. After 72 h, the monomer conversion values increased from 52% (50 °C) to 100% (80 and 90 °C), while *M*_n_ values increased from 760 g/mol (50 °C) to 1220 g/mol (90 °C). The optimal temperature of AFSET for the hydrolysis reaction has been determined to be 80 °C [42], however, its catalytic activity at 90 °C was even higher than at 80 °C in organic solvents. Thus, the intrinsic thermal tolerance of the AFEST enzyme and further stabilization through covalent attachment made the immobilized enzyme more suitable for polyester synthesis under harsh reaction conditions. Following this, the effect of reaction time on monomer conversion and *M*_n_ values was studied at 80 °C in toluene (Figure 4). 

Clearly, both monomer conversion and *M*_n_ values increased with the increase in reaction time: the monomer conversion reached 100% and did not show any changes with increased reaction time after 72 h; *M*_n_ exhibited a slightly increasing tendency, still in the range of 850–1250 g/mol. Thus, 72 h was selected as the optimal reaction time for the further PCL synthesis. In the non-aqueous medium, solvents played a key role in the stability of biocatalyst and the partition of substrates and products between the solvent and biocatalyst [43,44]. Here, a series of solvents with different log P values were employed as reaction media to evaluate the activity and stability of immobilized enzyme (Table 1). Like the free enzyme and immobilized enzyme EC-OD-AFEST [19,28], higher monomer conversion (>95%) and *M*_n_ values (940–1160 g/mol) were obtained in relatively hydrophobic solvents (toluene, cyclohexane and *n*-hexane). These results were probably caused by the favorable retention of essential water molecules on the enzyme’s surface in the hydrophobic solvents, which was beneficial for maintaining the catalytic conformation and activity of the enzyme. Additionally, the covalent immobilized enzyme EC-EP-AFEST could also be applied in solvent-free system, with monomer conversion and *M*_n_ values of 82% and 1050 g/mol. The solvent-free system shows great potential as an environmentally friendly PCL synthesis process, owing to its mild reaction conditions without using organic solvents and toxic catalysts. 

### 2.3. Operational Stability Analysis

The operational stability and recyclability of the immobilized enzyme were evaluated through a series of consecutive ring-opening polymerizations of ε-caprolactone as follows: 80 mg immobilized enzyme EC-EP-AFEST, 200 μL ε-caprolactone and 600 μL toluene at 80 °C for 72 h. 

**Figure 5:**
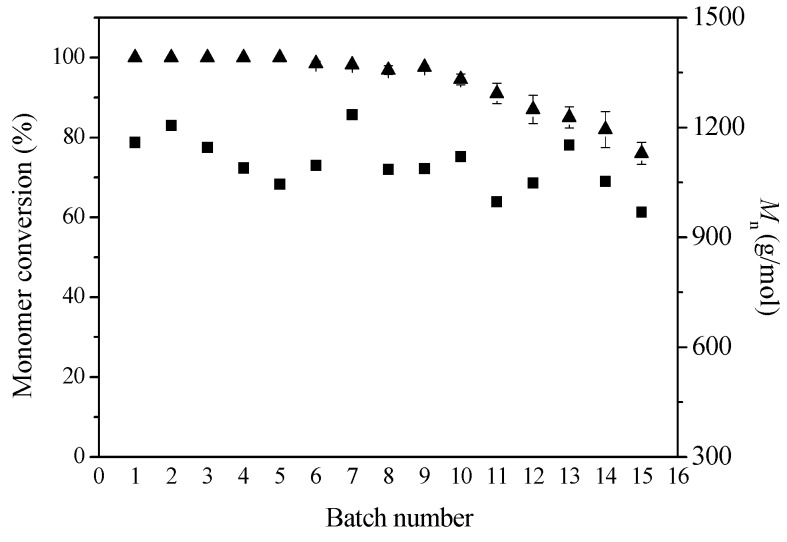
Monomer conversion and *M*_n_ values for a series of consecutive batch reactions. Reactions were conducted at 80 °C for 72 h, using 80 mg immobilized enzyme EC-EP-AFEST, 200 μL ε-caprolactone and 600 μL toluene.

After each reaction, the immobilized enzyme was recovered by filtration, washed with dichloromethane to remove any residual substrate and product, and then reused in the next batch reaction. As shown in Figure 5, the immobilized enzymes possessed good operational stability, with monomer conversion values higher than 75% and relatively stable *M*_n_ values (900–1200 g/mol) during 15 batch reactions. Compared to enzyme immobilized via physical adsorption on the hydrophobic macroporous resin Octadecyl-Sepabeads EC-OD [28], the immobilized enzyme prepared through covalent attachment possessed much higher operational stability and reusability. Meanwhile, due to the intrinsic higher activity and stability of the thermophilic esterase, the covalent attachment did not affect its catalytic efficiency in polyester synthesis. 

### 2.4. Gram-Scale Synthesis of PCL Using Immobilized Enzyme EC-EP-AFEST

**Figure 6:**
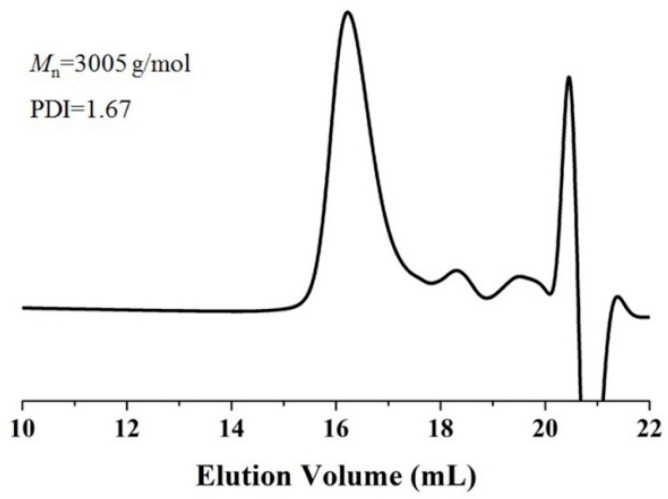
GPC chromatogram of PCL obtained in a gram-scale synthesis using immobilized enzyme EC-EP-AFEST. Reactions were conducted at 80 °C for 72 h, using 5 mL ε-caprolactone, 15 mL toluene and 2.0 g EC-EP-AFEST.

To demonstrate the practicality of the immobilized enzyme EC-EP-AFEST, a gram-scale synthesis of PCL was performed, with an isolated yield of 75%. After the structural characterization through ^1^H-NMR (Figure S1), the *M*_n_ and PDI values were determined to be 3005 g/mol and 1.67 using GPC analysis (Figure 6). Compared with the above research, the molecular weight showed an improved tendency due to the increased reaction scale, which was consistent with our previous report that PCL with *M*_n_ of 41,500 g/mol could be obtained by increasing the reaction scale in CALB-catalyzed ring-opening polymerization of ε-caprolactone [45]. However, the PDI value also increased, indicating a broad molecular weight distribution of the PCL. 

## 3. Materials and Methods

### 3.1. Materials

The recombinant *Escherichia coli* BL21 harboring the thermophilic esterase gene AF1716 from *A. fulgidus* was kindly donated by Dr. Giuseppe Manco (Istituto di Biochimica delle Proteine, Naples, Italy). Epoxy-Sepabeads EC-EP was kindly provided by Resindion S.R.L. (Mitsubishi Chemical Co., Binasco, Italy). ε-Caprolactone (>99%) was purchased from Sigma-Aldrich (Milwaukee, WI, USA) and used as received. Yeast extract and tryptone were purchased from Oxoid Ltd. (Basingstoke, UK). Organic solvents of analytical grade were purchased from Beijing Chemical Co. (Beijing, China), and dried over 4 Å molecular sieves from Tianjin Chemical. Co. (Tianjin, China) before use. All other chemicals were purchased with the highest reagent grade and used without further purification. 

### 3.2. Immobilization of Thermophilic Esterase on Epoxy-Sepabeads EC-EP

The recombinant thermophilic esterase AFEST was purified from the recombinant *E. coli* BL21 strain according to our previous research [19,28,46]. For enzyme immobilization, Sepabeads EC-EP (0.5 g) was washed with ethanol and distilled water, and then suspended in enzyme solution (100 mL, 1 mg/mL, 50 mM phosphate buffer (pH 8.0)) at room temperature for 24 h. The adsorption process was monitored by measuring the enzymatic activity in the supernatant. Afterwards the solid materials were filtered, washed with 50 mM phosphate buffer (pH 8.0) and lyophilized to obtain the immobilized enzyme EC-EP-AFEST. Finally, the enzyme loading on the support (mg AFEST/g support) was calculated, and the surface morphologies of carriers and immobilized enzymes were observed through scanning electron microscope (SEM, Micron PEI Philips, Hillsboro, OR, USA) with an accelerating voltage of 20 kV. The enzymatic activity was measured using *p*-nitrophenyl caprylate as the substrate at 80 °C [47], and one unit of esterase activity was defined as the protein amount releasing 1 μmol *p*-nitrophenol in one minute. 

### 3.3. Enzymatic PCL Synthesis

Standard enzymatic ring-opening polymerization of ε-caprolactone was conducted as follows: 200 μL ε-caprolactone, 600 μL organic solvent (with no addition of organic solvents in the solvent-free system), 50 μL of butyl acetate (internal standard) and different amounts of immobilized enzyme EC-EP-AFEST were mixed together in a screw-capped vial. During the reactions (stirring at 180 rpm), an aliquot of reaction mixture (10 μL) was taken via a syringe at regular intervals, diluted with dichloromethane (100 μL) and then analyzed by gas chromatography (GC) to determine the monomer conversion. After the reactions, dichloromethane was added to the systems, and enzymes were removed via filtration and washed with dichloromethane three times. The filtrate was collected and evaporated under reduced pressure to remove the dichloromethane. Following this, the viscous sample was precipitated in methanol at −20 °C, and the white precipitate was collected by the centrifugation at 8000 rpm for 15 min, which was then dried in a vacuum oven and identified by ^1^H-NMR in chloroform-d at 500 MHz on an AVANCE DMX 500 spectrometer (Bruker, Rheinstetten, Germany).

### 3.4. Determination of Monomer Conversion

The determination of monomer conversion was conducted on a 2014 gas chromatograph (Shimadzu, Tokyo, Japan) equipped with an Rtx-1 capillary column (30 m × 0.25 mm × 0.25 μm) and a hydrogen flame ionization detector, using nitrogen as the carrier gas. The temperatures were set as follows: injection pool (200 °C), detector (240 °C), and the column temperature was held at 70 °C for 2 min, and then increased to 140 °C at 10 °C/min and maintained for 2 min. The injection volume was 1.0 μL. A typical GC chromatogram is shown in Figure S2.

### 3.5. Determination of M_n_ and PDI

The *M*_n_ and PDI values of products were measured by gel permeation chromatography (GPC), in which polystyrene of narrow molecular weight distribution was used as standard. The assays were conducted on a Shimadzu HPLC system equipped with Shim-pack GPC-804 and GPC-8025 ultrastyragel columns and a refractive index detector, with tetrahydrofuran as the eluent (1.0 mL/min). The products were diluted in tetrahydrofuran at a concentration of 0.3% (*w*/*v*) and filtrated through 0.22 μm-micropore film. The injection volume was 20 μL. Typical GPC chromatograms are shown in Figure S3. 

### 3.6. Operational Stability Assay

The operational stability assay of immobilized enzyme was examined in repeated reuse as follows: 200 μL ε-caprolactone, 600 μL organic solvent and 80 mg EC-EP-AFEST were reacted at 80 °C for 72 h. The immobilized enzyme was recovered by filtration after each reaction, washed with dichloromethane and then reused for the next batch reaction. 

### 3.7. Gram-Scale Synthesis of PCL

To verify the practicality of using the immobilized enzyme in PCL synthesis, a gram-scale synthesis of PCL was conducted as follows: 5 mL ε-caprolactone, 15 mL toluene and 2.0 g EC-EP-AFEST were reacted at 80 °C for 72 h. The PCL product was extracted and structurally characterized using ^1^H-NMR in chloroform-d as described in Section 3.3. The GPC analysis to determine *M*_n_ and PDI values was performed on a Malvern 515 HPLC apparatus (Malvern, UK) with a Malvern 410 RI detector and T500 and T1000 columns. Tetrahydrofuran was used as the eluent at a flow rate of 1.0 mL/min at 35 °C. The detection was calibrated with polystyrene standards of narrow molecular weight distribution.

## 4. Conclusions

In the present research, covalent attachment was employed to construct an immobilized thermophilic esterase from the archaeon *A. fulgidus*, and then the immobilized enzyme was successfully applied in the synthesis of PCL. Compared to the free enzyme and enzyme immobilized via physical adsorption, the immobilized enzyme EC-EP-AFEST exhibited higher thermostability and better reusability. Thus, the immobilized enzyme possesses great potential for the mild, metal-free synthesis of polyesters, especially at an industrial scale. 

## Figures and Tables

**Table 1:** Monomer conversion and *M*_n_ values in various organic solvents and solvent-free system at 80 °C for 72 h.

Solvent	Log P	Monomer Conversion (%)	*M*_n_ (g/mol)	PDI
Dioxane	−1.10	26	n.d. ^1^	n.d.
Acetone	−0.23	29	n.d.	n.d.
Tetrahydrofuran	0.49	45	850	n.d.
Chloroform	2.00	60	970	1.19
Toluene	2.50	100	1160	1.21
Cyclohexane	3.09	95	940	1.22
*n*-Hexane	3.50	96	1110	1.22
Solvent-free	—	82	1050	1.23

^1^ n.d.: not determined.

## Supplementary Materials

The following are available online at http://www.mdpi.com/1420-3049/21/6/796/s1, Figure S1: ^1^H NMR spectrum of PCL synthesized by the immobilized enzyme EC-EP-AFEST; Figure S2: Typical GC chromatograph for determining the monomer conversion; Figure S3: Typical GPC chromatograms of polystyrene standard with *M*_n_ = 2000 g/mol (A) and PCL synthesized by the immobilized enzyme EC-EP-AFEST (B).

## Abbreviations

The following abbreviations are used in this manuscript:

PCL	Poly(ε-caprolactone)
*M*_n_	Number-average molecular weight
PDI	Polydispersity index
SEM	Scanning electron microscope
AFEST	*Archaeoglobus fulgidus* esterase
GC	Gas chromatography
CDCl_3_	Chloroform-d
GPC	Gel permeation chromatography
