# Supplementary material for: Construction of an Immobilized Thermophilic Esterase on Epoxy Support for Poly(ε-caprolactone) Synthesis
Source: Molecules. 2016 Jun 18;21(6):796. doi: 10.3390/molecules21060796 (PMC6272972; doi:10.3390/molecules21060796)
Supplement: Supplementary file 1 [file molecules-21-00796-s001.pdf]

SupplementaryMaterials: Construction of an Immobilized Thermophilic Esterase on Epoxy Support for Poly(ε-caprolactone) Synthesis

Hui Ren, Zhen Xing, Jiebing Yang, Wei Jiang, Gang Zhang, Jun Tang, Quanshun Li

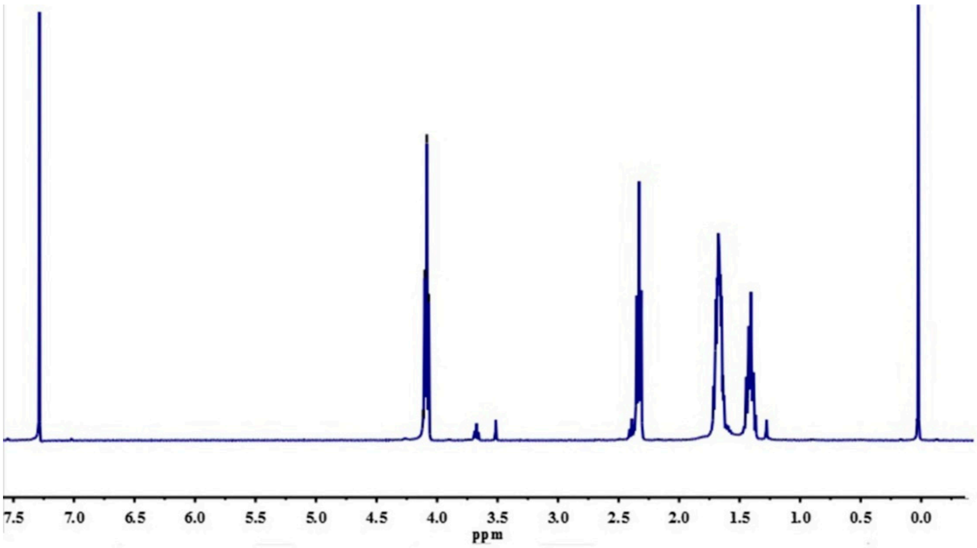

Figure S1. ^{1}H NMR spectrum of PCL synthesized by the immobilized enzyme EC-EP-AFEST. Reactions were conducted at 80 °C for 72 h, using 5 mL ε-caprolactone, 15 mL toluene and 2.0 g EC-EP-AFEST. ^{1}H-NMR of PCL: 1.39 (m, -COCH$_2$CH$_2$C*H*$_2$-), 1.66 (m, -COCH$_2$C*H*$_2$CH$_2$C*H*$_2$CH$_2$O-), 2.31 (t, -COC*H*$_2$-), 4.06 (t, -C*H*$_2$O-) and 3.66 (t, -C*H*$_2$OH end group).

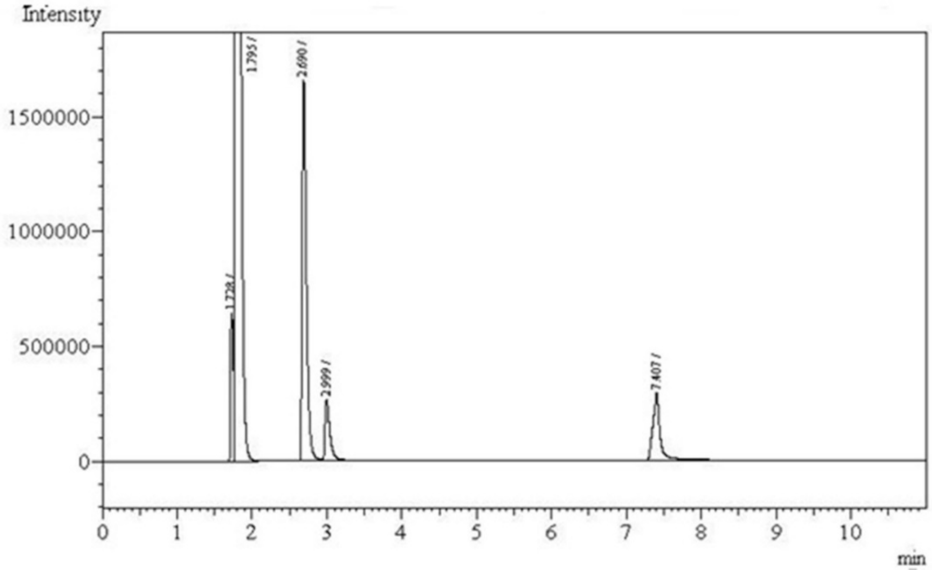

Figure S2. Typical GC chromatograph for determining the monomer conversion. The peaks at 1.795, 2.690, 2.999 and 7.47 min represented dichloromethane, toluene, butyl acetate (internal standard) and ε-caprolactone, respectively.

(A)

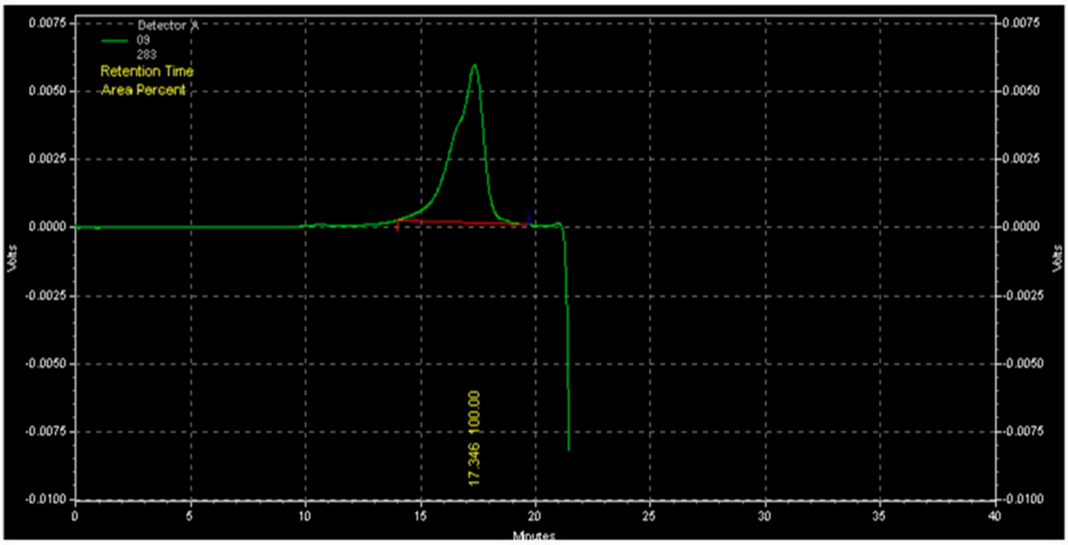

(B)

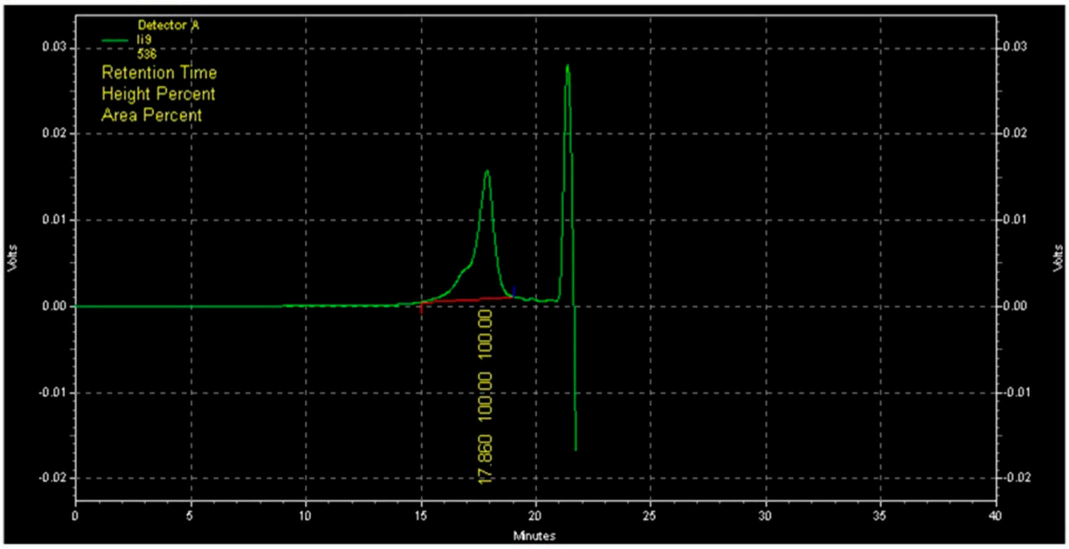

Figure S3. Typical GPC chromatograms of polystyrene standard with M_n = 2000 g/mol (**A**) and PCL synthesized by the immobilized enzyme EC-EP-AFEST (B).
